# 912. Expansion of Hepatitis C Treatment with New Medicaid Subscription Model in Louisiana

**DOI:** 10.1093/ofid/ofab466.1107

**Published:** 2021-12-04

**Authors:** Lauren E Richey, Yussef Bennani, Maria Frontini

**Affiliations:** 1 Louisiana State University, New Orleans, LA; 2 LSU Health Sciences Center - New Orleans, New Orleans, Louisiana; 3 Louisiana State University Health Sciences Center, New Orleans, Louisiana

## Abstract

**Background:**

It is estimated that nearly 80,000 people with hepatitis C are living in Louisiana, many with Medicaid coverage. Previously, only Medicaid patients free from drugs and alcohol with a fibrosis score of F3 or F4 were eligible for treatment, resulting in few patients receiving treatment. Beginning in July 2019, generic sofosbuvir/velpatasvir was made available through the Medicaid program in a subscription model, allowing unlimited hepatitis C treatment in Louisiana’s Medicaid program for 5 years at a set price to the program. This has dramatically expanded access to Hepatitis C treatment for people with Medicaid in Louisiana.

**Methods:**

Patients with Hepatitis C seen in the Infectious Diseases Center at University Medical Center in New Orleans, in 2020 by the 5 main hepatitis C providers were included. Demographics and laboratory data were collected to determine outcomes.

**Results:**

Most patients with a hepatitis C (HCV) viral load and insurance data had Medicaid (80%, N=275). Twenty-two (8%) were HIV co-infected. Most were men (75%) and African-American (77%). Among the mono-infected patients with Medicaid and an HCV viral load, 216 (85%) had an undetectable viral load by the beginning of June 2021. Of the remaining 37 patients, 30 patients were prescribed treatment; but did not take it (n=4), didn’t follow-up (n=23), or followed-up but never got labs (n=3). One was treated but had a treatment failure (n=1). Six of the 37 were not prescribed medications due to a short life expectancy or significant drug interactions. The percentage of patients with an undetectable viral load was similar by gender and race, however younger age groups had lower viral suppression. In those aged less than 35, only 47% had an undetectable viral load and among those aged 36 to 44, it was 66%. Using the previous criteria of requiring a fibrosis score of F3 or F4, only 20% (n=44) would have been eligible for medicine to treat hepatitis C.

**Conclusion:**

The new hepatitis C treatment subscription model with resultant removal of previous barriers has dramatically expanded treatment for people with Medicaid in Louisiana. More than five times the number of Medicaid patients received treatment in 2020 in our academic medical clinic.

**Disclosures:**

**Yussef Bennani, MD, MPH**, **Gilead Sciences** (Scientific Research Study Investigator)**ViiV Healthcare** (Scientific Research Study Investigator)

